# Inhibitory control training reveals a common neurofunctional basis for generic executive functions and language switching in bilinguals

**DOI:** 10.1186/s12868-021-00640-5

**Published:** 2021-05-17

**Authors:** Yan Jing Wu, Mo Chen, Guillaume Thierry, Yongben Fu, Junjie Wu, Taomei Guo

**Affiliations:** 1 State Key Laboratory of Cognitive Neuroscience and Learning, Beijing Normal University, Beijing, 100875 People’s Republic of China; 2 Faculty of Foreign Languages, Ningbo University, Ningbo, China; 3 School of Psychology, Bangor University, Bangor, Gwynedd UK; 4 IDG/McGovern Institute for Brain Research, Beijing Normal University, Beijing, China

**Keywords:** Inhibitory control, Language production, Language control, Transfer effect, fMRI

## Abstract

**Background:**

The neural networks underpinning language control and domain-general executive functions overlap in bilinguals, but existing evidence is mainly correlative. Here, we present the first neurofunctional evidence for a transfer effect between (domain-general) inhibitory control and language control through training. We trained Chinese–English bilinguals for 8 days using a Simon task taxing the inhibitory control system, whilst an active control group was trained with a color judgment task that does not tax the inhibitory control system. All participants performed a language-switching task before and after training. It has been suggested that the activity of the left DLPFC was associated with domain-general top-down cognitive control (Macdonald et al. Science 288: 1835–1838, 2000) and bilingual language control (Wang et al. Neuroimage 35: 862–870, 2007). In addition, the dACC was closely related to the conflict detection (Abutalebi et al. Cereb Cortex 18:1496–1505, 2008). Last, the activity of the left caudate has been linked with lexical selection (Abutalebi et al. Cereb Cortex 18:1496–1505, 2008), especially the selection of the weak language (Abutalebi et al. Cortex 49: 905–911, 2013). Therefore, we focused on these three regions of interest (ROIs) where neural changes associated with transfer were expected to occur.

**Results:**

The results showed a negative correlation between changes in activation levels in the left dorsolateral prefrontal cortex (DLPFC) and changes in the switch cost magnitude in the language-switching task in the training group but not in the control group, suggesting that the DLPFC plays a critical role in the transfer effect from domain-general executive functions to language control. However, there was no measurable effect in the anterior cingulate cortex or left caudate nucleus, suggesting that the inhibitory control training increased the neural efficiency for language production in bilinguals in terms of attention shifting and conflict resolution, but the training did not affect conflict detection and lexical selection.

**Conclusion:**

These findings showed how cognitive training evidence can help establish a causational link between the neural basis of domain-general executive functions and language control in bilinguals.

## Background

Bilingual individuals activate both languages when using one of their two languages (e.g., [19, 20, 34, 38, 76]). Therefore, they need a control mechanism to prevent interference from the non-target language to the target language [31]. Previous studies have mainly used the language-switching paradigm to examine language control in bilinguals (e.g., [21, 55, 62]). In a classic language-switching paradigm, bilingual participants name pictures or digits in their native language (L1) and their second language (L2) according to the language cue. In each trial, participants either name the current stimulus in the same language as in the previous trial (non-switch trial) or in the other language (switch trial). In a seminal study, Meuter and Allport [62] showed greater switch costs, that is, the difference in reaction time or error rate between switch and non-switch trials, in forward switches (i.e., from L2 to L1) as compared to backward switches (i.e., from L1 to L2) switches. This finding is consistent with assumptions of the Inhibitory Control Model [31]: Unbalanced bilinguals have to inhibit the dominant language (L1) to a greater extent as compared to the non-dominant language (L2) when switching between the two languages, and thus forward switches are more costly than backward switches. The asymmetry in switch cost has been replicated by numerous studies (e.g., [21, 55, 68]) and the switch cost magnitude is widely regarded as an index for language control capacity in bilinguals.

Functional neuroimaging studies have shown that language switch in bilinguals activates a neural network comprising of the left dorsolateral prefrontal cortex (DLPFC), the dorsal anterior cingulate cortex (dACC), the supplementary motor area, and the left caudate nucleus (LCN; [1, 4, 13, 25, 33, 36, 37, 54, 71, 78, 79]). Some of these brain regions, such as the DLPFC and dACC, have been shown to be critical structures for the domain-general inhibitory control (IC) system i.e., the ability to prevent interference from task-irrelevant information [66]. Given that these regions are also involved in language processing, the IC system is thought to neurofunctionally overlap with the language control system [4, 24, 80]. Consistent with this view, bilinguals with below average IC capacity experience greater language switch costs [50–52] and make more cross-language errors (e.g., they produce the incorrect language more often) as compared to bilinguals with higher IC capacity [32].

Short-term training using IC tasks such as the Go/No-go task and the Simon task has been shown to improve behavioral performance and to concurrently modulate underlying neural activation patterns [11, 12, 16, 17, 49, 58, 59]. Some studies have also shown that improvements in specific IC training tasks can transfer to untrained tasks [10, 41, 60, 64]. For example, training using the Simon task correlatively improves behavioral performance in the flanker task, suggesting that the two tasks might tap onto similar underlying cognitive components and share the underlying neural substrates [23]. In the same view, given the overlap between the IC system and the language control system introduced above, one could also expect a transfer between IC training and language control in bilinguals. To test this hypothesis, Liu et al. [51] engaged Chinese-English bilinguals in a six-day training program with a modified version of the Simon task. Event-related brain potentials (ERPs) showed that the IC training increased the difference between native (L1) and second (L2) language in the mean amplitude of late positive component when performing the language-switching task, suggesting an enhanced inhibitory control of L1 during L2 production, especially during lexical response selection phase. Moreover, the transfer effect was stronger in participants with relatively lower IC capacity as compared to those with higher IC capacity, indicating an interaction between training transferability and individual differences in inhibitory control.

**Fig. 1:**
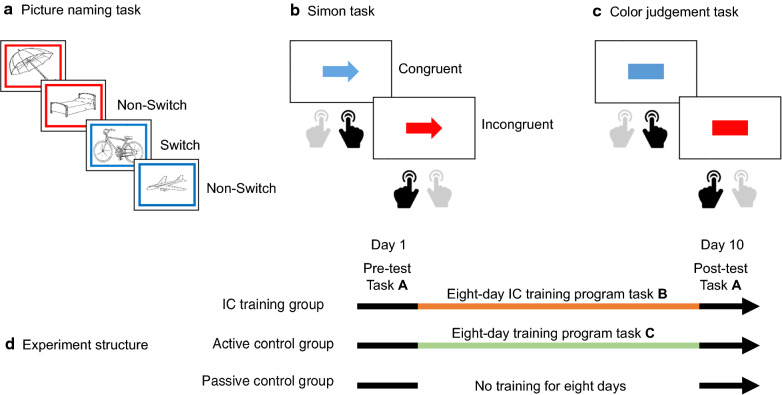
Tasks and procedure used in the present study. **a** Sample stimuli from the picture naming task (“name picture in Chinese if the frame is red, or in English if the frame is blue”); **b** Sample stimuli and expected responses in the Simon task (“press button on side indicated by blue arrow, press on opposite side of that indicated by red arrow”); **c** Illustration of the color judgement task (“press left button for red or right button for blue”); **d** Protocol of the study. All participants were pre-tested on the first day and post-tested on the tenth day. The Inhibitory Control (IC) training group and the active control group received an eight-day training program involving the Simon task or the color judgment task, respectively, while the passive control group received no training between pre- and post-test sessions

To our best knowledge, the neural mechanisms underlying the transfer effect between IC and language control in bilinguals have not been specified. In a previous study, we showed that short-term training with language switching reduced activation in the dACC and LCN [43]. Here, we aim to characterize the neural substrates underpinning transfer from IC training to language control. Sixty-six Chinese-English bilinguals were randomly assigned to the training group, the active control group, or the passive control group. The training group engaged in an eight-day training programme with a modified version of the Simon task (e.g., [53, 82, 83]), the active control group engaged in an eight-day training programme with a color judgment task that does not tap onto inhibitory control; and the control group did not receive any training between the pre- and post-training testing sessions (Fig. 1). To quantify transfer effects from IC training to language control, all three groups performed a language-switching task before and after the training while hemodynamic fluctuations were monitored using functional magnetic resonance imaging (fMRI; e.g., [43, 44, 82, 83]).

**Fig. 2:**
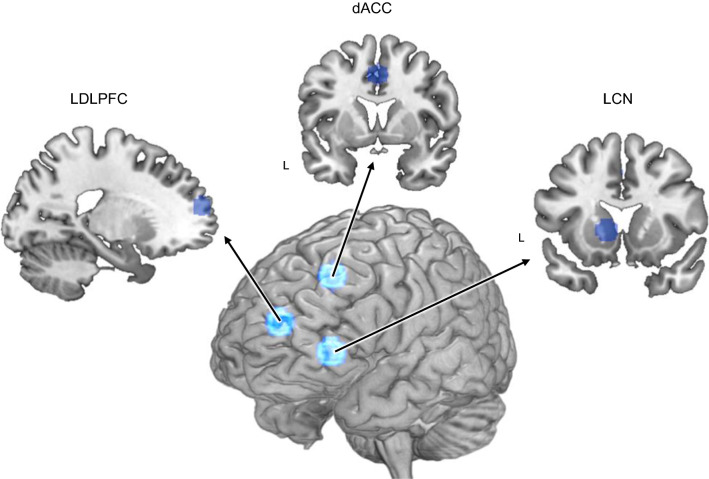
D3 visualization of the three regions of interest (ROIs): left dorsal lateral prefrontal cortex (DLPFC) (MNI coordinate: x = -18, y = 58, z = 17), dorsal anterior cingulate cortex (dACC) (MNI coordinate: x = 0, y = 6, z = 44), and lateral caudate nucleus (LCN) (MNI coordinate: x = -11, y = 15, z = 1). All ROIs were 8-mm radius spheres

We targeted regions of interest (ROIs) in three brain regions where neural changes associated with transfer were expected to occur: the dACC, the left DLPFC, and the left caudate nucleus (LCN, Fig. 2).

Previous studies have shown that dACC, left DLPFC, and LCN play distinct roles in language control during language production in bilinguals [2]. The dACC is thought to be involved in detecting cross-language competition, while the left DLPFC modulates levels of attention and resolves overcoming cognitive conflicts [2]. The LCN, however, is thought to play a critical role in language-specific lexical selection [1, 4], although the LCN has also been implicated in domain-general action selection and executive control (see [14], for a review). Given that the Simon task taps onto two main cognitive processes, namely, attentional modulation and conflict monitoring, our hypothesis is that IC training would affect activation levels in all three ROIs. However, with regard to the training effect on language switching, the prediction is that the training group would show significant neurophysiological changes in the left DLPFC and dACC, but not in the LCN, since DLPFC and ACC are related to IC while the LCN is associated with processing language-specific information. Finally, we expected the active and passive control group would not show any training effect.

## Methods

### Participants

Sixty-six Chinese-English bilinguals participated in the present study. They all had normal or corrected-to-normal vision, and reported no neurological conditions or cognitive deficits. Participants were randomly assigned to either the inhibitory control (IC) training group, the active control, or the passive control group. Seven participants were excluded from the data analysis due to excessive head movement (i.e., > 3 mm) during the fMRI scan. As a result, the IC training group included 20 participants, the active control group had 19 participants, and the passive control group had 20 participants.

All participants took the College English Test Band 4 (CET4), which is the national English test for college students in China, obtaining an average score of 516 (*SD* = 54, the maximum score on the test being 710). Participants were thus relatively proficient rather than highly proficient in English. In addition, all participants rated their proficiency in Chinese and English on a 10-point scale with a higher score indicating higher proficiency. The 3 (Group: IC training/Active control/Passive control) × 2 (Language: Chinese/English) repeated measures ANOVA performed on the self-rating scores showed that the main effect of language was significant, *F*(1, 56) = 179.51, *p* < 0.001, *ƞ*_*p*_^*2*^ = 0.762, while the main effect of group and the interaction were not, *F*s < 1, indicating that Chinese was the dominant language for all three groups, and that language proficiency was matched for both Chinese and English across groups. The training group, the active control group and the passive control group did not differ in terms of age, sex, AOA, L2 proficiency measured by CET-4, and fluid intelligence (*p*s > 0.1, see Table 1).

**Table 1:** Participant characteristics

	IC training group (*N* = 20)	Active control group (*N* = 19)	Passive control group (*N* = 20)	*F* value/Chi^2^ value	*p* value
Age	21.9 (2.0)	22.4 (2.1)	22.6 (2.5)	0.46	0.634
Sex	12 females	10 females	13 females	0.70	0.706
Age of L2 acquisition (AOA)	10.1 (2.4)	8.5 (2.6)	9.4 (2.5)	1.80	0.175
L1 self-rating score	8.2 (1.2)	8.4 (1.2)	8.4 (1.1)		
L2 self-rating score	5.3 (1.4)	5.3 (1.1)	5.9 (1.2)		
CET-4 score	516 (52.1)	525 (54.8)	507 (55.0)	0.51	0.604
Fluid intelligence	56.5 (3.5)	56.5 (3.7)	55.1 (3.7)	1.09	0.344

L1: the first language; L2: the second language

### Stimuli

Forty black-and-white line drawings were selected from the database of Snodgrass and Vanderwart [75] for the formal experiment, while the other eight pictures were used as the practice items. These pictures were also used in some of our previous studies (e.g., [82, 83]. Each picture was presented at 12.5 cm wide and 9.5 cm high in the center of the screen subtending a vertical visual angle of approximately 4.9° and horizontal visual angle of 6.5°.

### Procedures

The study was approved by the Ethical Committee of the State Key Laboratory of Cognitive Neuroscience and Learning at Beijing Normal University. Written informed consent was obtained from each participant prior to participation. The experiment included a pre-test session, a training session, and a post-test session (see Fig. 1).

In the pre-test session, participants first performed a practice picture naming task (e.g., [82, 83]). At the beginning of each trial, a fixation cross was presented for 300 ms. Then, a picture was presented within a colored frame in either red or blue for 1000 ms. There was 200 ms interval between the fixation and the picture. Participants were required to name the picture using the language as indicated by the language cue (i.e., the frame color) as quickly and accurately as possible in a soft voice. The inter-trial interval (ITI) was 1 s, 2 s, 3 s, or 4 s. The association between cue and naming language was counterbalanced across participants. The practice task included 16 trials. The formal experiment included two runs. Each run included 82 trials and lasted 5 min and 28 s. Owing to the unstable magnetic field at the beginning of a scan, the first two trials (i.e. first four scans) of the formal experiment were excluded from statistical analyses. The remaining 80 trials were orthogonally manipulated between the trial type (switch/non-switch) and language (Chinese/English) with 20 trials for each condition in each language. The whole scanning session lasted about 25 min, including an 8-min anatomical scan at the end.

Due to technical limitations (i.e., the lack of MRI-compatible microphones), behavioral data were collected outside of the scanner. To reduce carryover effects, participants returned to the experiment a few hours after the fMRI session and performed the same task in a behavioral lab. At the end of the pre-test session, all participants filled out a basic information form and performed the Raven’s Progressive Matrices [72] to obtain information about their language learning history and fluid intelligence.

After the pre-test, the IC training group received an eight-day training session involving domain-general IC training for approximately 30 min every day. The training task was a modification of the classic Simon task (e.g., [53, 82, 83]). At the beginning of each trial, a 200-ms fixation cross was presented at the center of the screen. Then, a colored arrow pointing either to the left or to the right was presented after a 300 ms blank screen. Participants had to respond by pressing one of two buttons with their left or right hand. The arrow was then replaced by a blank screen for 1500–2000 ms (i.e., the ITI). The color (blue or red) of the arrow specified the condition of the trial. For example, when the arrow was blue, participants had to press the button on the same side to which the arrow pointed (i.e., the congruent condition), when the arrow was in red, participants had to press the button on the opposite side to which the arrow pointed (i.e., the incongruent condition). One training session included six blocks, each containing 81 trials. The first trial was excluded from analyses. The remaining trials included 40 congruent trials and 40 incongruent trials. To increase the task difficulty, the correspondence between the color of the arrow and the congruency of responses was reversed between blocks. The training procedure was identical for each day of the eight-day training program.

The active control group received 8-day training on color judgment for approximately 30 min every day. The procedure of this task was identical to that of the Simon task, expect that participants were asked to judge the color of a rectangle (i.e., no specific directions presented in the stimuli). Participants were instructed to press the left (or right) button when the rectangle was blue (or red). The correspondence between the color of the rectangle and the responses was reversed between blocks.

All participants took the post-test session on the tenth day after the eight-day training program. The post-test session was identical to the pre-test session except that anatomical scans were not obtained in the post-test session.

### Data collection

Functional and anatomical images were captured by a 3-T Siemens Sonata MRI scanner. All participants were required to lay on the scanner and fixed with a 12-channel coil to avoid head motions. Functional scans were acquired using a T2-weighted echo-planar imaging (EPI) sequence. 33 axial slices were collected with an interleaved acquisition order. The parameters were set as follows: TR = 2000 ms, TE = 20 ms, flip angle = 90°, FOV = 200 × 200 mm^2^, matrix size = 64 × 64, resolution within slices = 3.1 × 3.1 mm^2^, slice thickness/gap = 4 mm/0.8 mm, and number of slices = 164. The high-resolution T1-weighted anatomical images were also obtained. The parameters were set as follows: TR = 2530 ms, TE = 3.39 ms, flip angle = 7°, FOV = 256 × 256 mm^2^, matrix size = 256 × 256, resolution within slices = 1.0 × 1.0 mm^2^, slice thickness = 1.33 mm, and number of slices = 144.

### Behavioural data analysis

Trials with a reaction time below 200 ms or above 1500 ms (0.4% in the IC training Group, 6% in the active control group, and 2% in the passive control group) were excluded from the analysis as outliers [44]. In addition, we excluded reaction times more than 2.5 standard deviations below or above each participant’s mean value (2% in the IC training Group, 1% in the active control group, and 2% in the passive control group). Statistical analyses were then performed on the remaining correct trials.

### Imaging data analysis

Data preprocessing and whole-brain analyses were performed using the SPM 8 toolbox (The Wellcome Centre for Human Neuroimaging, UCL Queen Square Institute of Neurology, London, UK). We discarded the first four images to allow magnetization to reach the equilibrium state for each participant. The remaining images were entered into final analyses. Slice-timing correction and realignment were firstly performed. The realignment parameters were then examined to check head motion. Seven participants were excluded due to large head motions (absolute motion greater than 3 mm). The remaining images were then normalized to the T1 template and resliced with 3 × 3 × 3 mm voxels. The images were finally smoothed by a cubic FWHM = 6 Gaussian kernel.

At the individual level, a General Linear Model was used to estimate the contrast of interest for each participant. Statistical analyses were performed by modeling different conditions on a voxel-by-voxel basis. The data were globally scaled and high-pass-filtered at 128 s. The contrast of interest at the individual level was the difference between switch trials and non-switch trials in the pre-test session and post-test session.

At the group level analysis, we performed a 3 (Group: IC training/Active control/Passive control) × 2 (Trial type: Switch/Non-switch) ANOVA on the three groups in the pre-test session. To avoid false positive results, only clusters with at least 15 contiguous voxels activated above the threshold of *p* < 0.05 (FDR corrected) were considered significant.

For the region of interest (ROI) analyses, we defined three brain regions according to coordinates reported in previous studies. It has been suggested that the activity of the left DLPFC was associated with domain-general top-down cognitive control [57] and bilingual language control [78]. We defined the left DLPFC ROI according to the coordinate (− 18, 58, 17) reported by Wang et al. [78]. Coordinates were converted from the Talairach space to the MNI space using the algorithm developed and validated by Jack Lancaster [48]. In addition, the dACC was closely related to the conflict detection [1]. We defined the dACC ROI, which showed significant activation for language control, according to coordinates (0, 6, 44) reported by Abutalebi et al. [3]. Last, the activity of the LCN has been linked with lexical selection [1], especially the selection of the weak language [4]. We defined the LCN ROI according to the average of coordinates (− 11, 15, 1) reported by Abutalebi et al. [1], Abutalebi et al. [4], and Zou et al. [86]. All ROIs were 8-mm radius spheres. We then extracted the beta values of the switch cost (i.e. switch trials minus non-switch trials) for each ROI at an individual level. The locations of ROIs are illustrated in Fig. 2.

Finally, to further examine the relationship between transfer effects on the neural mechanisms and behavioral performance, Pearson correlation analyses of the correlation between the neural changes in beta values of the switch cost (i.e. switch cost in pre-test session minus that in post-test session) and behavioral changes in switch costs after training were conducted for each ROI.

## Results

### Behavioral results

**Fig. 3:**
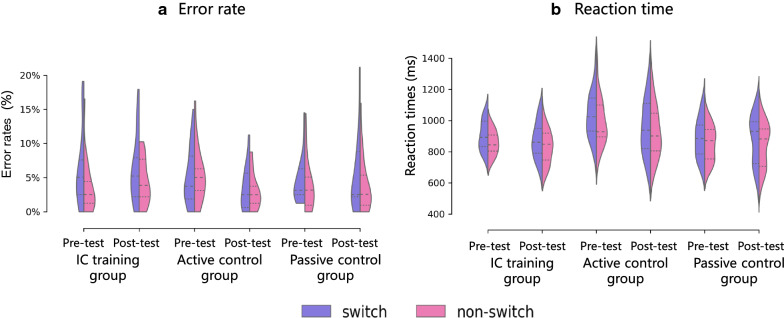
Error rates and naming latencies in experimental and control groups in pre- and post-test sessions

The 3 (Group: IC training/ Active control/ Passive control) × 2 (Test session: Pre-test/ Post-test) × 2 (Trial type: Switch/ Non-switch) repeated measures ANOVA conducted on error rates revealed a main effect of trial type, *F* (1, 56) = 17.50, *p* < 0.001, *ƞ*_*p*_^*2*^ = 0.238, such that switch trials led to more errors than non-switch trials (Fig. 3a).

The interaction between trial type and group was significant, *F* (2, 56) = 5.36, *p* = 0.007, *ƞ*_*p*_^*2*^ = 0.161. Bonferroni corrected follow-up comparisons showed that individuals in the IC training group were less accurate in switch trials than non-switch trials, *F* (1,56) = 24.58, *p* < 0.001, *ƞ*_*p*_^*2*^ = 0.305. The difference between switch and non-switch trials was marginally significant in the passive control group, *F* (1,56) = 4.02, *p* = 0.050, *ƞ*_*p*_^*2*^ = 0.067 and non-significant in the active control group, *F* (1,56) < 1.

The interaction between group and test session was significant, *F* (2,56) = 4.50, *p* = 0.015,* ƞ*_*p*_^*2*^ = 0.139. Bonferroni corrected follow-up comparisons showed that individuals in the active control group performed more accurate in the post-test than in the pre-test, *F* (1,56) = 9.68, *p* = 0.003,* ƞ*_*p*_^*2*^ = 0.147. There was no significant change in the IC training group (*F* (1,56) < 1) and passive control group ( *F* (1,56) < 1).No other significant main effect or interactions were found (Test session: *F* (1,56) = 1.46, *p* = 0.232,* ƞ*_*p*_^*2*^ = 0.025; Group: *F* (2,56) < 1; Test session × Trial type:* F* (1,56) < 1; Group × Test session × Trial type:* F* (2,56) = 1.34, *p* = 0.271,* ƞ*_*p*_^*2*^ = 0.046).

The 3 (Group: IC training/ Active control/ Passive control) × 2 (Test session: Pre-test/ Post-test) × 2 (Trial type: Switch/ Non-switch) repeated measures ANOVA conducted on reaction times (RTs) showed a significant main effect of trial type, *F* (1, 56) = 102.68, *p* < 0.001, *ƞ*_*p*_^*2*^ = 0.647, indicating a significant switch cost (Fig. 3b).

The main effect of test session was also significant, *F* (1, 56) = 16.41, *p* < 0.001, *ƞ*_*p*_^*2*^ = 0.227, indicating that participants were overall faster in the post-test than the pretest session. The main effect of group was significant, *F* (2, 56) = 4.74, *p* = 0.013, *ƞ*_*p*_^*2*^ = 0.145. Further analysis (Bonferroni corrected) revealed that RTs were slower in the active control group than in the IC training (*p* = 0.028) and passive control (*p* = 0.030) groups, but the training group and passive control groups did not differ between them (*p* = 1). The interaction between group and test session was significant, *F* (2, 56) = 4.58, *p* = 0.014, *ƞ*_*p*_^*2*^ = 0.1. Further analysis showed that both the IC training group and the active control group tended to respond faster after training (IC training group: *F* (1,56) = 3.86, *p* = 0.054,* ƞ*_*p*_^*2*^ = 0.065; active control group: *F* (1,56) = 21.00, *p* < 0.001,* ƞ*_*p*_^*2*^ = 0.273), but there was no significant improvement in the passive control group (*F* (1,56) = 0.17, *p* = 0.683,* ƞ*_*p*_^*2*^ = 0.003). No other significant interactions were found (Group × Trial type:* F* (2,56) = 0.715, *p* = 0.494,* ƞ*_*p*_^*2*^ = 0.025; Test session × Trial type:* F* (1,56) = 0.59, *p* = 0.446,* ƞ*_*p*_^*2*^ = 0.010; Group × Test session × Trial type:* F* (2,56) = 1.01, *p* = 0.370,* ƞ*_*p*_^*2*^ = 0.035).

### Whole brain results

**Fig. 4:**
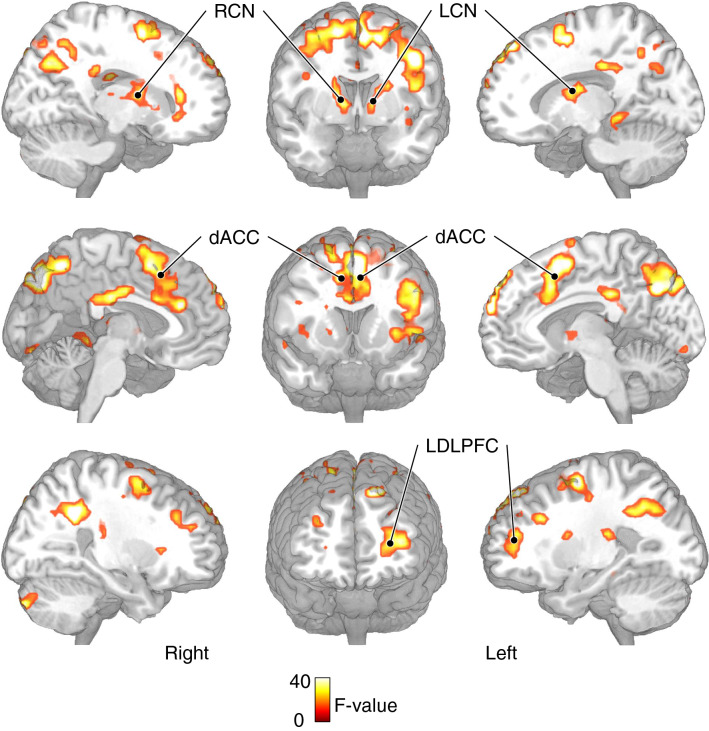
Activation maps for the main effect of trial type (Switch > Non-switch) (p < 0.05, FDR corrected, k > 15)

The 3 (Group: IC training/ Active control/ Passive control) × 2 (Trial type: Switch/Non-switch) repeated measures ANOVA performed on the three groups in the pre-test session showed significant main effect of trial type in the left medial superior gyrus, the left supplementary motor area and critically, the left DLPFC, but also the left inferior parietal lobule, the left precuneus, the right orbital inferior frontal gyrus, the right anterior and middle cingulate cortex, the right cerebellum, and bilateral caudate nucleus (see Table 2 and Fig. 4). However, the main effect of group and the interaction between group and trial type were not significant in any of the brain regions.

**Table 2:** The brain areas with significant activation for the main effect of trial type revealed by the 3 (Group: IC training/ Active control/ Passive control) × 2 (Trial type: Switch/ Non-switch) repeated measures ANOVA (FDR corrected, p < 0.05, cluster corrected, *k* > 15)

Brain regions	BA	Cluster size	MNI coordinates (x, y, z)			*F-*value
L medial superior frontal gyrus	10	86	− 6	66	24	32.47
L caudate nucleus	–	80	− 24	6	18	17.84
L supplementary motor area/DLPFC	6	463	− 3	15	54	38.25
L precuneus	7	197	− 9	− 66	36	31.59
L inferior parietal lobule	40	80	− 33	− 48	36	28.28
R orbital inferior frontal gyrus	13	27	48	21	− 9	21.23
R caudate nucleus	–	115	12	3	3	18.28
R anterior cingulate cortex	32	25	3	36	21	24.99
R Middle cingulate cortex	23	18	6	− 21	27	20.35
R cerebellum		19	33	− 87	− 39	28.58

BA: Brodmann area; L: left; R: right

### ROI results

**Fig. 5:**
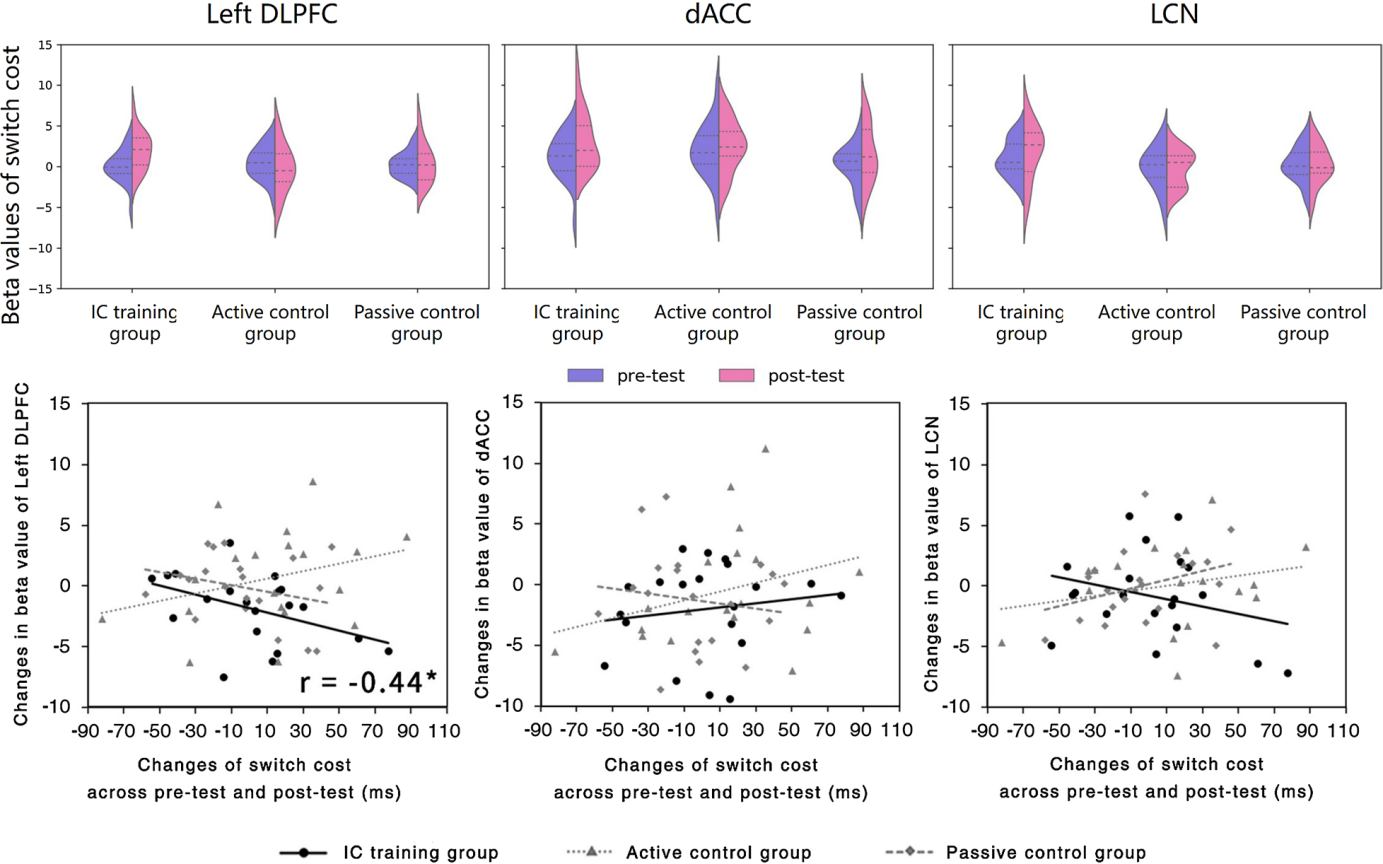
ROI results. Top panel: beta values of switch cost in each of the three ROIs (left DLPFC, dACC, and LCN) at pre-test and a post-test. Beta values increased significantly in the left DLPFC only in the IC training group. Beta values of switch cost increased significantly in the dACC in all three groups. Bottom panel: correlation between the change in beta value of switch cost and change in switch cost between test sessions

In the DLPFC, a 2 (Test session: Pre-test/Post-test) × 3 (Group: IC training/Active control/Passive control) ANOVA of beta values did not validate a main effect of test session, *F*(1, 56) = 1.11, *p* > 0.2,* ƞ*_*p*_^*2*^ = 0.019, or a main effect of group, *F*(1, 56) = 1.73, *p* = 0.187,* ƞ*_*p*_^*2*^ = 0.058, (Fig. 5). However, the interaction between test session and group was significant, *F*(2, 56) = 3.17, *p* < 0.05, *ƞ*_*p*_^*2*^ = 0.102. Bonferroni corrected follow up tests showed a significant increase in beta values between post- and pre-test session, in the IC training group, *F*(1, 56) = 6.71, *p* = 0.012,* ƞ*_*p*_^*2*^ = 0.107, but such differences did not reach significance in either the active control or the passive control groups (*Fs*(1, 56) < 1).

For the dACC, the ANOVA showed a significant main effect of test session, *F*(1, 56) = 5.27, *p* < 0.05, *ƞ*_*p*_^*2*^ = 0.086, suggesting that activation in the ACC was stronger after training. The main effect of group was not significant, *F*(1, 56) = 2.28, *p* > 0.1, *ƞ*_*p*_^*2*^ = 0.075 and neither was the interaction between group and test session, *F*(2, 56) < 1.

For the LCN, the ANOVA showed that the main effect of test session was not significant, *F*(1, 56) < 1. The main effect of group was significant, *F*(1, 56) = 3.75, *p* < 0.05, *ƞ*_*p*_^*2*^ = 0.118. Post hoc analysis (Bonferroni corrected) showed significant difference between IC training group and active control group, *p* < 0.05, but no other overall difference in beta values between groups (IC training group vs. passive control group:* p* = 0.179; active control group vs. passive control group:* p* = 1). The interaction between group and test session was not significant, *F*(2, 56) < 1.

### Correlation results

A correlation analysis showed that the change in the beta values associated with switch costs (i.e., the contrast between switch and non-switch trials) in the left DLPFC was negatively correlated with the change in the behavioral switch costs,* r* = − 0.440, *p* = 0.026, in the IC training group (Fig. 5. No such correlation was found in the dACC (*r* = 0.150,* p* = 0.264) or LCN (*r* = − 0.283,* p* = 0.113) in the same group. In the two control groups, we found no significant correlation between beta value change and switch cost change in any of the three ROIs (active control group: DLPFC (*r* = 0.307,* p* = 0.100), dACC (*r* = 0.310,* p* = 0.098), or LCN (*r* = 0.253,* p* = 0.148); passive control group: DLPFC (*r* = − 0.279,* p* = 0.117), dACC (*r* = -0.145,* p* = 0.270), or LCN (*r* = 0.335,* p* = 0.075).

## Discussion

The present study examined transfer effects of domain-general, non-linguistic cognitive training to the neural correlates of language control in Chinese-English bilinguals. To start with, fMRI results showed that the language-switching task activated a neural network including left medial superior gyrus, the left supplementary motor area including the DLPFC, the left inferior parietal lobule, the left precuneus, and the right orbital inferior frontal gyrus, the right anterior and middle cingulate cortex, the right cerebellum, and bilateral caudate nucleus. Previous studies have shown that these brain regions are involved in language control (e.g., [78, 79], [24, 29, 54, 84, 85]). Our results are therefore in accordance with the view that language control in bilinguals involves a widely distributed neural network, including cortical, subcortical, and cerebellar areas [5].

As expected, fMRI results showed no significant difference between the training group and the control groups in the pre-test session, suggesting that the language control ability, and its underlying neural mechanisms, were comparable between the three groups prior to training. In contrast, the ROI analysis showed that IC training increased activation levels of the left DLPFC associated with language-switching cost in the IC training group, but the two control groups showed no such effect. The left DLPFC has been implicated in top-down attentional modulation and conflict resolution [9, 63, 65, 69, 74]. It is also involved in resolving cross-language competition and interference in bilingual language production [1, 25, 33, 36, 37, 78, 79]. Our findings, therefore, showed a neurofunctional transfer effect from short-term IC training to the domain of language control, indicating that IC training strengthens attentional modulation and competition resolution when bilinguals switch between languages.

Moreover, there was a significant negative correlation between changes in the activation levels of the left DLPFC and the changes of switch costs in the behavioral performance of the training group. That is, individuals with smaller switch costs at the post-test showed greater changes in left DLPFC activation levels. This finding provides direct evidence for a functional link between language control performance and the neuroplasticity in the left DLPFC. Indeed, previous studies have shown that training increases activation levels in brain regions supporting cognitive control [26, 28, 35, 39, 67]. As proposed by Kelly and Garavan [46], training-induced neuroplasticity is a flexible, complex, and dynamic process that might lead to functional reorganization of a cluster of neural substrates independent of activation levels in a specific brain region. It has been suggested that, unlike associations between reduced neural activity and enhanced functional efficiency, higher neural activation levels as a result of training might reflect in-depth changes in the neurophysiological underpinning of the cognitive operations trained [45, 46, 70]. One possible explanation for the transfer effect found in the present study is that extensive practice with a simple task increases task-processing efficiency through automatic transfer effects (i.e., abstract transfer effects, [81]. In the present study, cue congruency correspondence was altered frequently in the Simon task during IC training to enhance the training intensity. As a result, participants in the training group might have recruited more neural resources during cue detection when performing the language-switching task. This explanation is consistent with findings that far transfer effects, which reflect positive gains from one domain to a distal domain, are associated with increased neural activation levels [18, 23, 73].

Interestingly, activation levels in the dACC were increased in the post-test session as compared to the pre-test session in all three groups of participants. The dACC has been associated with domain-general conflict detection and monitoring [6, 27, 47, 51]. Studies have also shown that bilinguals recruit the dACC to monitor cross-language interference [1, 4, 25, 33, 78, 79]. One possibility is that the increased dACC activation observed in all three groups might be a result of task repetition rather than a training effect, because the IC training group and active control group had different training procedure, and the passive control group received no training at all. Prior studies have shown functional dissociations between the dACC and DLPFC. While activation in the ACC have been associated with conflict evaluation and monitoring, the DLPFC is associated with attentional allocation and top-down cognitive control [15] and plays a critical role in conflict resolution [30]. With regard to language control, Abutalebi et al. [1] proposed that the dACC is involved in the detection of language conflict and, through projection to the DLPFC, the dACC reinforces (i.e., raises the activation levels) of neural representations of the target language in order to resolve competition between the target and non-target language. Taken together, these findings provide new support for the hypothesis that domain-general IC training can enhance attentional modulation and conflict resolution during bilingual language production. In comparison, practices (i.e., the repetition effect) with the language-switching task itself enhance different domains of the language control system (i.e., detecting language competition).

As expected, LCN activation levels did not change significantly between the pre-test and post-test session in any of the three groups. Previous studies have shown that the LCN is activated when bilinguals perform language production tasks [1, 22, 71], but the role of LCN has been understood as processing language-specific information, such as lexical selection, rather than domain-general executive control [1, 4, 61]. For example, Abutalebi et al. [1] showed increased activity of the LCN when bilinguals named pictures in a dual-language context (i.e., where both languages are involved in the task and compete for lexical selection), but not in a single, native language context (i.e., no cross-language competition). The role of the LCN in lexical selection is also supported by lesion studies [7, 8]. In a recent study, Kang et al. [43] examined the neural plasticity of language control using a language-switching task. Unlike the task transfer manipulation in the current study, the same task was used in both the training and the testing sessions in Kang et al., [43] and the results showed that training with a language-switching task decreased neural activations in the LCN. This finding suggests an effect of language control training on the neural efficiency of the LCN. In the current study, the null result in the LCN is consistent with the fact that the Simon task used in the training programme did not harness the lexical selection mechanism and, therefore, had no effect on the activity of LCN associated with the language-switching task.

It should be noted that there was no transfer effect of IC training on behavioral performance in the language-switching task, despite that the behavioral results showed a typical switch cost before and after training for all three groups of participants. The switch cost is manifested as increased reaction times in switch compared to non-switch trials, a pattern that is consistent with previous studies on bilingual word production [55, 62]. This finding suggests that the language-switching task used in the present study was effective for measuring language control in bilinguals. Other studies [42, 56, 73, 77, 87] have also failed to observe significant behavioral effects associated with training. For example, in a series of studies on language-switching training, Kang et al. [43, 44] found that significant behavioral improvement was only observed when the exact same task and procedure was used for both the training and the testing session. Trivial alterations in experimental materials and parameters such as ISI between the language cue and the stimulus can lead to a null result in terms of behavioral performance enhancement. Other studies have shown that the absence of a behavioral improvement through training could also be explained by task difficulty, and the intensity and length of training [11, 59].

## Conclusion

In summary, it has been speculated a connection between domain-general inhibitory control and language control in bilinguals [24, 25, 50, 51, 80], but a causal link between the two systems have not been established. Here, for the first time, we demonstrated neurophysiological transfer between domain-general, non-linguistic cognitive training and key neuroanatomical structures at the heart of the language control system in bilinguals [1]. Given that the neuroplastic changes were observed in the left DLPFC and dACC, we suggest that the language control system shares three critical components with the domain-general IC system: attentional modulation, conflict detection, and conflict resolution. In contrast, the LCN which has been implicated in language-specific processing was not affected by the IC training programme. Therefore, by examining neural transfer effects of domain general inhibitory control training, our study provides new insights into the overlapping (i.e., left DLPFC and dACC) and dissociated (i.e., LCN) neural substrates that underpin the domain-general IC and language control systems, and specified the cognitive functions these distinct neural mechanisms are involved in. Given that language proficiency and individual variances in cognitive control ability may modulate the effect of cognitive training [40, 52], future studies can further investigate how these individual factors might affect with neural functional connections between the two systems.

## Data Availability

The datasets generated and analysed during the current study are available in the Mendeley repository, Chen, Mo (2020), “Inhibitory control training & language control”, Mendeley Data, V1, https://doi.org/10.17632/dysy7s6whb.1.

## Abbreviations

fMRI: Functional magnetic resonance imaging
DLPFC: Dorsolateral prefrontal cortex
dACC: Dorsal anterior cingulate cortex
LCN: Left caudate nucleus
L1: First language
L2: Second language
IC: Inhibitory control
ERPs: Event-related brain potentials
CET4: College English Test Band 4
SD: Standard deviation
AOA: Age of L2 acquisition
ITI: Inter-trial interval
ROI: Region of interest
RCN: Right caudate nucleus
